# Knowledge and perceptions of male immigrants in Leeds (UK) towards male circumcision as an HIV prevention strategy

**DOI:** 10.4102/sajhivmed.v20i1.823

**Published:** 2019-10-31

**Authors:** Catherine Atuhaire, Kabanda Taseera, Chris Spoor, Rosaline Y. Cumber, Samuel N. Cumber

**Affiliations:** 1 Department of Nursing, Faculty of Medicine, Mbarara University of Science and Technology, Mbarara, Uganda; 2 Faculty of Health Science, Leeds Beckett University, Leeds, United Kingdom; 3 Faculty of Political Science, University of Kwazulu-Natal, Durban, South Africa; 4 Section for Epidemiology and Social Medicine, Department of Public Health, Institute of Medicine, The Sahlgrenska Academy at University of Gothenburg, Gothenburg, Sweden; 5 Faculty of Health Sciences, University of the Free State, Bloemfontein, South Africa

**Keywords:** male circumcision, knowledge, perceptions, HIV prevention, United Kingdom

## Abstract

**Background:**

The World Health Organization has accepted and recommended medical male circumcision (MMC) as an HIV prevention strategy. Despite the advantages of MMC, the rate of uptake of this practice among immigrants and the general population in the United Kingdom (UK) is low, yet the procedure is provided in public and private health facilities. The role of negative perception and its contribution to low circumcision rates is unknown.

**Objectives:**

Since immigrants are a key group that is vulnerable to HIV in the UK, this study aimed at understanding their knowledge and perceptions with regard to MMC.

**Methods:**

We enrolled 10 participants who were purposively selected using snowball recruitment methods. Data were collected during individual in-depth interviews using semi-structured interview guides. Responses were audio recorded, transcribed and analyzed using thematic analysis. Appropriate themes were generated from the data collected.

**Results:**

We found that the majority looked at male circumcision (MC) as a practice to fulfill their cultural and religious obligations rather than as an HIV protection method. Few participants showed belief and certainty that MC or MMC was effective in HIV prevention hence limited knowledge. They also expressed perceived danger. This included fear of pain, complications from the procedure and possible infections when carried out through traditional means. These dangers discouraged study participants from accessing MMC.

**Conclusion:**

Male circumcision is mainly practiced to fulfill cultural and religious norms, but is not seen as a credible HIV prevention strategy.

## Introduction

Medical male circumcision (MMC) is a globally accepted and recommended HIV prevention strategy.^1,2^ However, the success of this strategy depends on its uptake within the community, something consequent upon cultural acceptance, knowledge and perceptions of its effectiveness in the prevention of HIV transmission.

In the United Kingdom (UK), the prevalence of HIV among immigrants, especially those from southern and eastern Africa, is higher than that of the indigenous population, namely 25 per 1000 men and 50 per 1000 women versus 2.1 per 1000 men and 1 per 1000 women, respectively.^3,4^ This is partly attributed to the high prevalence rates of HIV infection in the home countries of immigrants and, as some argue, to complacency with regard to HIV prevention.^5^

Behavioural change is an important instrument in the public health promotion of MMC. For change and acceptability to happen, people’s knowledge and perceptions have to change.^6^

We conducted in-depth interviews to assess the knowledge and perceptions of circumcision as an HIV prevention tool among male immigrants from southern and eastern Africa, but currently resident in the UK.

## Background

HIV is a major health challenge, especially in sub-Saharan Africa.^2^ In 2013, more than 35 million people worldwide were infected and 1.5 million died of HIV and AIDS.^7^ Male circumcision (MC), which is the surgical removal of the foreskin of the penis, supplements less effective strategies such as abstinence, faithfulness to a partner and the consistent use of condoms as a means to prevent infection.^8,9,10^ East and southern African countries have implemented MMC as a primary prevention strategy. The widespread introduction of MMC followed compelling evidence of the reduction of HIV acquisition in circumcised, uninfected and exposed men of ≥ 40%.^11^

The biological plausibility and efficacy of the procedure arises from the fact that the penile foreskin contains many CD4 receptor-bearing Langerhans cells and lymphocytes. These cells permit viral (HIV) invasion.^12^ In addition to the removal of vulnerable tissue, circumcision assists in increasing the thickness (keratinisation) of residual skin, thereby reducing penile abrasions during intercourse.^13^ Circumcision may reduce the incidence of genital ulcer disease and in this way also ‘protect’ from HIV infection.^14^

In order for rates of MC or MCC to increase, society and the individual’s perceptions must be better understood. Indeed, without perception, action, according to Gibson,^15^ is misguided and serves no purpose.^16^ The promotion of successful health strategies, for example MC and MMC, must influence people’s perceptions.^17^

Although the number of people diagnosed with HIV annually in the UK has been low, namely 5000 in 2001, the number of those living with HIV is increasing and by the end of 2011 was estimated to be 96 000.^1,11^ Disease is not restricted to a geographical region or a specific population group. To reduce or eradicate HIV from the UK, prevention strategies must focus on the needs of key and vulnerable populations such as immigrants. Given the political and economic instability of countries in Africa, the number of those seeking asylum in the UK is likely to grow.

The World Health Organization (WHO) has identified 14 countries in east and southern Africa with generalised HIV epidemics and with low prevalence rates of MC. These countries have been targeted for the scale-up of MMC programmes.^1^ In the UK, circumcision of British men is on the decline and currently stands at 15.8% of 16–44-year olds. Rates vary: high among Jewish men (98.7%), and lower among Hindus, Sikhs and Buddhists (9.8%).^18^ The overall low rate of circumcision in the UK likely follows on directives from the National Health System (NHS) that recommends circumcision only if medically indicated.^19^

The role of negative perception and its contribution to low circumcision rates is unknown. This study sought to better understand the knowledge base and perceptions of male immigrants from eastern and southern Africa now residing in Leeds (UK) with regard to the role of MC or MMC as an HIV prevention strategy.

## Materials and methods

### Study population and setting

A cross-sectional qualitative research approach was used for the evaluation of the knowledge and perceptions of male immigrants living in Leeds, the UK, and originally from southern and eastern Africa. All eligible participants had lived in the UK for more than 2 years and had a reasonable command of the English language. The study focused on participants from this part of Africa because of the high prevalence of HIV infection in their region of origin.^2,20^

### Tools for data collection

Data were gathered using semi-structured interview guides. The responses were audio recorded, although participants had been offered the option of having their responses hand written in case they felt uncomfortable with recording. Each interview took approximately 45–60 min.

### Data collection

Participants were purposively selected using a snowball recruitment method. Data were collected through face-to-face researcher-guided in-depth interviews. During the preliminary stage, an eligible person was identified. The subject was invited to participate in the study and given the Participant Information Sheet to read before consenting to participate. He was then contacted and invited to an interview. After the interview, this person was asked if he knew any potential participants from the region of Africa under study and was requested to pass on details to the researcher. This referral process was applied until the sample size was reached. To allow for response diversity, participants from different age groups and nationalities were contacted. The questions required the participants to define circumcision, any benefits and cultural views in support of or against MC or MMC. The research questions were formulated based on the study objectives, and each interview was allocated approximately 45–60 min. Data were collected in 2 months.

### Data management

Data were analysed using a thematic content analysis. In brief, transcripts were read and re-read several times. Initial codes were identified, noting repeated issues. Each code was checked against the raw data, and emerging ones were developed into categories. The categories were grouped together into overarching themes, based on the understanding of the data. Appropriate themes were generated and recorded. When checking the raw information, a description was provided that summarised the theme. These transcripts were analysed manually and checked for consistency.

This method of data analysis was used to describe in detail the views, opinions and feelings of respondents. Verbatim accounts have been used in the presentation of the data, and results compared with other similar studies so as to contextualise the work.

### Research rigour

An assessment of data trustworthiness was undertaken throughout this study that focussed on credibility, transferability, conformability and dependability.^21^

Credibility was ensured through establishing a conducive rapport with participants, while conformability was done by evaluating interview questions to ensure that they were not leading and closed-ended. Codes used for each participant allowed for proper matching of each participant’s description.

Dependability was ensured by careful listening to the recorded responses to explain phrases that were used by the participants and transferability was also achieved by eliciting descriptions of findings, adequate sampling and achieving data saturation.

### Limitations

Because this study targeted immigrants from southern and eastern Africa, the sampling was difficult, as it was not an easily identifiable population. The researcher had to resort to a snowball recruitment technique. Consequently, bias is possible as the sampling may have captured a group of like-minded friends. The researcher attempted to limit bias by recruiting respondents from different African countries. It is acknowledged that bias may remain as peers may share the same views or beliefs. The small number of participants is a further limitation as additional views and perceptions were excluded, that is, it is acknowledged that this study cannot represent the views of all citizens of east and southern Africa or of all African immigrants. However, the uniformity of the results suggests that the study has captured some important themes. Relevant additional demographic data such as the grouping of participants’ age and response, educational (highest educational level achieved) and socio-economic (employment) status were not included in the study, but may have influenced responses of the study members.

### Ethical consideration

The Leeds Metropolitan University approved the conduct of the study and informed consent was obtained from eligible participants. All ethical issues were addressed. Efforts were made to build trust and rapport with participants during the interview process. Potential risks to the participants included breach of confidentiality, possible sensitivity of the respondents to the research questions and a feeling of uneasiness towards the interviewer. To mitigate these risks, the participants received as much information as possible before the interviews, so that they decide whether or not to participate in the study.

## Results

A total of 12 informants were interviewed; however, the data analysis could be performed on only 10 (Table 1).

**TABLE 1:** Key informants’ demographics.

Variable	Frequency (*N* = 10)	%
**Gender**		
Male	10	100
**Marital status**		
Single	3	30
Married	5	50
In a relationship	2	20
**Countries of origin**		
Zimbabwe	3	30
Malawi	1	10
South Africa	2	20
Kenya	2	20
Uganda	2	20

All respondents were between 18 and 65 years of age, and had lived in the UK for more than 2 years. The various geographical backgrounds of the cohort, ages and relationships were thought to provide a wide range of views, opinions and perceptions. Results are presented thematically using verbatim reporting.

### Knowledge of male immigrants about male circumcision as an HIV prevention measure

To achieve this objective, respondents were asked what they understood MC or MMC was, the benefits and views of the relevance of MC or MMC in the prevention of HIV.

### Meaning of male circumcision

All the 10 participants were able to define MC:

… my understanding of circumcision is the removal of the foreskin from the penis of a man. That is my understanding … (Key Informant 2, male, married)

… I believe it’s the cutting of the foreskin of the male penis … (Key Informant 3, male, married)

Although all the 10 participants understood MC as removal of the foreskin from the penis, one respondent defined MC in a cultural sense:

… circumcision I believe is what certain cultures undergo as a culture … (Key Informant 4, male, single)

Some respondents understood circumcision from a religious point of view as one participant revealed:

… I mean am a Christian. Circumcision the way I see it was directed by God. Ok from the bible … … Genesis 18 verse 10 says this is my covenant you shall keep between me and you and thy seed after thee. Every male child among you shall be circumcised … I don’t see any medical basis for it. It is just a religious thing … (Key Informant 10, male, single)

The findings show that participants viewed circumcision as being done for medical, cultural and religious purposes.

### Benefits of male circumcision

We explored whether participants knew MC as an HIV prevention strategy. Responses to this question were spontaneous as we wanted to find how HIV prevention ranked among the benefits of MC. Participants gave various benefits of MC and HIV prevention ranked third.

### Male circumcision for cultural purposes

Culture has been suggested in various studies as both the determinant and predictor of MC. From spontaneous responses, culture was viewed as the major benefit of MC. Out of 10 respondents, six mentioned culture as the reason for MC and that the procedure helped fulfil cultural obligations and fitted in with the accepted cultural practices:

… Firstly, some cultures tend to practice that. When a child is born if he is male, at a certain age he has to get circumcised. It is a way of passing from being a boy into manhood … (Key Informant 3, male, married)

… this is my understanding. Perhaps if you are not circumcised, how do you say you are a man because for us we believe that to be a man fully, you have to undergo a knife … (Key Informant 5, male, married)

… well in South Africa boys in certain tribes especially in the Eastern Cape, are expected to go through an initiation into manhood and this initiation starts with circumcision because they believe that the foreskin is a feminine part of the body and so this is removed … (Key Informant 8, male, single)

For most of the participants, circumcision is one way a boy can transit from childhood to adulthood. Fulfilling cultural obligations seemed to be the major benefit provided by MC.

### Perceptions towards male circumcision

Although MC or MCC as an HIV prevention strategy was the core of this study, this aspect was third in importance in the view of respondents and was mentioned by only four subjects. However, only one gave this as of first importance. Another mentioned it as of second importance after culture, and two placed it fourth after culture, religion and hygiene:

… if you remove the foreskin you also protect yourself from STIs and in the same manner I think circumcision helps HIV prevention. It is not 100% to my understanding but it’s to quite a good degree, it helps people from contracting HIV … (Participant 2, male, married)

The above participant was the only one who mentioned HIV prevention as the first benefit with some level of certainty. In some cases, participants were aware that MC or MCC protects against HIV, but were not sure whether it was true as revealed below:

… Am also told that one is that in the HIV perspective. I don’t know how true it is … (Participant 4, male, single)

… I have heard that it helps in HIV/AIDS prevention and other people do it for religious beliefs, I think … I just read it in one of the magazines that it helps people in HIV prevention but am not sure in the technical details … (Participant 1, male, in a relationship)

Two, however, revealed that MC protects against sexually transmitted infections (STIs) but could not mention which:

‘… really, to me, well I feel, it helps to prevent some sexually transmitted diseases … all the sexually transmitted diseases. Most of them, you can name them …’ (Participant 7, male, married)

So based on this study, it was clear that the level of knowledge about MC as an HIV prevention strategy was low, with cultural and religious factors taking precedence over HIV prevention.

### Social factors besides HIV prevention

In this study, participants revealed that MC had a social importance. Three participants mentioned that MC can help a person fit in his society or peer group:

… Hmm social aspect, I think as with most things really, many people are taking decisions based on the society. I think if you look at an individual, one is defined by where they belong to a group of people who uphold circumcision then you may want to get circumcised … (Participant 3, male, married)

The issue of social benefits was also echoed by other participants, showing that it was an important determinant of MC decisions:

… I think it is accepted in society, you know. In society, if you are not circumcised, you cannot really fit in. you know, discriminating and all that … (Participant 7, male, married)

… yes, but it is not something done because of medical reasons. Much as it is not done for those reasons, in the long run it will help protect an individual from acquiring STIs. It is quite beneficial if it is medical male circumcision … (Participant 2, male, married)

Therefore, based on such views, the decision to undergo MC or MCC or not can be a social decision, not necessarily to prevent HIV.

### Sexual satisfaction

Furthermore, one participant linked MC to sexual satisfaction revealing that being circumcised gave one increased sexual pleasure:

… you really get the feeling of, I wouldn’t call it satisfaction but when you are into sex … in my own view, it is true. … I mean during sexual intercourse. A man can run for longer hours which is advantageous to the women because they enjoy it more … (Participant 5, male, married)

Five participants revealed that they were aware that MC or MMC prevents HIV, but were not sure of how this was possible:

… I understand because I read a lot. I understand that there is a link between and it is being practiced in Uganda where they are encouraging most people to get circumcised as a prevention of HIV. How it is or the specifications, am not sure … (Participant 4, male, single)

One participant revealed that he had heard of the link, but could not believe it was possible and showed scepticism:

… We have heard always in media but specifically I have not yet believed. (Participant 5, male, married)

One participant did not have any idea that MC or MCC had any link to HIV prevention:

… Well I don’t know what exactly the link is. I only know studies that have looked at the link between HIV and circumcision. … I don’t know if there is any proof exactly how it could prevent HIV … (Participant 8, male, single)

To achieve objective 2, participants were also asked what they thought were the disadvantages or dangers of MC or MMC.

Pain came up prominently as one of the barriers to circumcision, as was mentioned by five participants:

… First and foremost, I would say to human beings, pain is something that is not easy to persevere so perhaps I do think they would fear the pain … (Participant 5, male, married)

This study found that people do not only fear to face the knife, but also fear possible complications that may arise out of the procedure, especially if it is done traditionally:

… Hmm I would like to believe that circumcision is a surgical and there is bound to be complications especially traditionally. If you are undergoing through circumcision and then there are complications that could be a disadvantage … (Participant 3, male, married)

Lastly, some participants believed that circumcision of males can actually lead to HIV infection instead of protecting against it, especially when done traditionally:

… understanding is that they just use the same instrument to cut all the boys. And there is no sterilising of instruments. Because of that I think those are the disadvantages … (Participant 1, male, in a relationship)

Responses presented show that although there is some awareness of MC or MCC as an HIV prevention strategy, most people still view the procedure from a cultural and a social point of view. Even among the people who are aware of the positive relevance of MC in HIV prevention, there is limited knowledge of how effective the strategy is, resulting in scepticism.

## Discussion

The efficacy of any health strategy relies on how the target population is aware of the strategy and the benefits it can provide. This was made evident in a study carried out^22^ in Botswana, where before the informational session, 68% of respondents were willing to circumcise their male children, but the percentage of those willing to circumcise their children increased to 89% after the session. This study sought to establish the knowledge regarding MC or MCC as an HIV prevention strategy. Our study found that there were still gaps in the knowledge of immigrants from east and southern Africa. Male circumcision is still largely looked at in the light of culture (five participants) and religion (five participants), rather than HIV prevention.

Regarding culture, other studies have also tried to explain how culture informs health choices and behaviour. Shweder suggested that MC often seems to be a social phenomenon, propelled by the need for individuals to fulfil cultural norms and practices, that enables males to acquire the traits of masculinity.^23^ Such cultures are embedded in African societies like the Gisu and Bakonjo in Uganda, Kikuyu of Kenya, Masai of Kenya and Tanzania. In the modern era, the cultural aspect of MC remains in regions such as east and southern Africa. However, in the majority of these areas, most of the rites that used to accompany such ceremonies are no more. In many cases, the procedure is now performed in private, for example one-on-one, in a hospital or doctor’s surgery. Local pain relief is often used in such settings. However, this modification is not acceptable to all tribes.^24^ The antagonists of modernisation insist on circumcision within a group ceremony, without anaesthesia, and as a test of courage at the banks of a river. This traditional approach is common among the Meru and Kissi tribes of Kenya.^24^ Despite the loss of the traditional appeal of circumcision, the physical effects are crucial to personal identity, pride and acceptance in society. Uncircumcised men in such communities risk being banished, and subject to ridicule as if they were boys. There have been many reported cases of forced circumcision of men from such communities, who are discovered to have escaped the ritual. Culture as the primary reason for MC in this study is largely explained by the fact that the participants came from countries where some cultures continue to practise circumcision.

The findings showed that there was uncertainty regarding MC or MCC as an HIV prevention strategy. Even after concerted efforts by the WHO to promote this,^2,25^ this study suggests that many individuals do not agree. The findings concur with Naidoo et al.,^26^ who suggested that even among educated people such knowledge is limited. The latter study assessed university students in Kwazulu-Natal, South Africa.

Based on the findings, it is evident that although there has been increased information, especially through the media and peer groups about MMC, little has been done to help people understand how MC protects against HIV. As a result, scepticism about the appropriateness of the procedure remains. This explains why educated people seem to be more likely to accept MC as an HIV prevention strategy (participant 1). This was demonstrated in a study carried out in Uganda^27^ and Kenya,^28^ where it was found that levels of awareness were higher among educated adults in rural areas.

Male circumcision is often associated with genital hygiene (participants 4, 7, 8). It is believed that when a man undergoes MC or MMC, hygiene is guaranteed. A study by Hill et al.,^29^ from Papua New Guinea, found that genital hygiene was the second most frequent reason given in support of MC.

This study suggests that adult male migrants from east and southern Africa may be unlikely to recommend MMC and opt rather for a view in line with culture, religious and social expectations.

## Conclusion

This study explored the knowledge and perceptions of male immigrants from southern and eastern Africa with regard to MC or MMC as an HIV control measure. The study indicates that the understanding of many immigrants is defective in this respect. It is suggested that attempts to increase awareness of the benefits of MMC be encouraged and that MMC be made more accessible and affordable to the immigrant community in the UK.
